# New progress in pediatric allergic rhinitis

**DOI:** 10.3389/fimmu.2024.1452410

**Published:** 2024-09-16

**Authors:** Miao Cheng, Qianqian Dai, Zhi Liu, Yulin Wang, Cuiyun Zhou

**Affiliations:** ^1^ Department of Ophthalmology and Otolaryngology, Jingmen Centra Hospital, Jingmen Central Hospital Affiliated to Jingchu University of Technology, Jingmen, Hubei, China; ^2^ Department of Infectious Disease, Jingmen Central Hospital, Jingmen Central Hospital Affiliated to Jingchu University of Technology, Jingmen, Hubei, China; ^3^ Department of Pediatrics, Zhongnan Hospital of Wuhan University, Wuhan, Hubei, China; ^4^ Department of Pediatrics, Jingmen Central Hospital, Jingmen Central Hospital affiliated to Jingchu University of Technology, Jingmen, Hubei, China

**Keywords:** allergic rhinitis, children, allergen immunotherapy, health management, trained immunity

## Abstract

The prevalence of allergic rhinitis (AR) in children is steadily increasing, and its onset is closely associated with genetic factors, living environment, and exposure to allergens. In recent years, an increasing number of diagnostic methods have been employed to assist in diagnosing AR. In addition to pharmaceutical treatments, personalized approaches such as environmental control and allergen-specific immunotherapy are gradually gaining popularity. In this article, we reviewed recent research on the etiology, diagnostic classification, treatment methods, and health management of AR in children. These insights will benefit the implementation of personalized diagnosis and treatment for children with AR, promoting health management strategies that improve symptoms and quality of life.

## 1 Introduction

Allergic rhinitis (AR) is a chronic non-infectious inflammatory disease of the nasal mucus mainly mediated by IgE, triggered by exposure to allergens (1). AR is among the most prevalent chronic diseases globally and is the leading chronic disease in children in the United States (2, 3). It is estimated that approximately 500 million people globally suffer from AR symptoms, leading to substantial economic burden and health impacts. The primary clinical manifestations of AR include rhinorrhea, nasal congestion, nasal pruritus, and sneezing (4). Although AR symptoms may appear mild, their impact should not be underestimated in children. Approximately 20% of children experience AR symptoms by ages 2 to 3, about 40% by age 6, and roughly 30% during adolescence. AR can significantly affect sleep, emotional well-being, cognitive function, and productivity in both work and study environments (5). In children, the impact of AR on quality of life is often more subtle compared to adults, frequently leading to fatigue, reduced attention span, impaired learning, and memory, which are sometimes overlooked or misinterpreted by parents as behavioral issues (6).

Currently, the awareness of AR in children among healthcare providers and the families of affected children remains insufficient. The quality of life of children with AR continues to be impacted by delayed or improper treatment. Treatment methods such as environmental control measures and allergen-specific immunotherapy (AIT) have garnered significant attention. Additionally, health management strategies utilizing mobile communication technology to collect data and guide treatment offer new perspectives for managing AR in children. This review addresses the epidemiology, Etiology, diagnosis, classification, treatment, and management of AR in children. It aims to enhance the understanding of healthcare providers and families, improve diagnostic accuracy and timeliness, and promote appropriate treatment and adherence.

## 2 Epidemiology

Data from the International Study of Asthma and Allergies in Childhood (ISAAC), which includes multi-center data from over 300 countries worldwide, showed that AR often begins in early life, with a prevalence of over 5% at the age of 3, 8.5% at 6-7 years, and increasing to 14.6% at 13-14 years (7). In the 13-14 age group, the incidence of AR is 9.2% in Northern and Eastern Europe, 18% in Africa, 17.3% in Latin America, and as high as 51% in the United Arab Emirates (8). These data suggest that the prevalence of AR in children increases with age and varies significantly across different regions.

It is noteworthy that due to the limited self-reporting ability of infants and young children, AR in children is prone to be overlooked by parents or mistakenly treated as non-AR, especially in lower-income areas, where this phenomenon may be more pronounced (9, 10). A recent meta-analysis indicates an approximately 22% increase in the prevalence of AR among Chinese children in recent years, with significant regional variations and higher incidence rates in industrially developed cities (11). Furthermore, the reported prevalence of childhood AR by family members may be higher than the actual level (12). A survey conducted in Wuhan, China, revealed a self-reported AR prevalence of 28.6% among 6-12-year-old children, whereas the doctor-diagnosed prevalence of AR in children aged 0-17 was 14.4% (13). Therefore, strengthening health education on AR within families may serve as a measure to reduce treatment delays resulting from inadequate awareness and to mitigate overtreatment due to an overestimation of symptoms.

## 3 Etiology

The primary cause of AR in children is allergen exposure. Outdoor allergens include pollen and mold, while indoor allergens comprise dust mites, animal dander, insects, and mold. Additionally, genetic susceptibility, family history of allergies (such as AR, asthma, and atopic dermatitis), antibiotic use, and passive smoking are factors associated with the risk of developing AR in children (14, 15). The occurrence and progression of AR depend on the interaction between genetic predisposition and environmental factors.

In 1989, British scholar Strachan first reported a negative correlation between the prevalence of hay fever in children and both family size and the number of older siblings in the household. This observation led to the formulation of the hygiene hypothesis which posits that fewer infections in early childhood increase the likelihood of developing allergic diseases later in life (16). The underlying mechanism of the hygiene hypothesis is that microbial antigens promote Th1 responses and inhibit Th2 responses. This aligns with the pathogenesis of AR, where an imbalance between Th1/Th2 immune responses leads to the release of inflammatory mediators by effector cells such as mast cells, basophils, and eosinophils, causing inflammation of the nasal mucosa.

Recent studies explored the impact of the hygiene hypothesis on the development of pediatric AR by investigating the diversity of “microbial burden” in early life and its relationship with allergic predisposition (17). For example, a Polish study found that children who started kindergarten at age two had double the risk of developing AR compared to those who started at age one (18). Similarly, Han et al. identified that not having pneumonia in early childhood and shorter playtime were risk factors for AR in Korean children (19). A recent meta-analysis involving over two million subjects also reported that higher birth order and a greater number of siblings were associated with a lower risk of AR (20). Early kindergarten attendance, larger family size, higher birth order, and longer playtime may indicate earlier or more frequent exposure to pathogens. Additionally, some studies have shown that reduced gut microbiota diversity in newborns, due to factors such as antibiotic use, is related to the development of AR in children (21). Although these studies support the hygiene hypothesis, these factors only roughly reflect early microbial exposure in children and lack more direct and robust evidence.

The hygiene hypothesis, while influential, falls short in comprehensively explaining the pathogenesis of AR. It attributes the development of allergic diseases primarily to a lack of pathogen exposure, overlooking other critical factors such as genetics, environmental influences, and dietary habits. Family history-based studies underscore the significance of genetics in AR, with genome-wide association studies (GWAS) in recent years identifying numerous genetic susceptibility loci and candidate genes (22, 23). For instance, the interleukin-4 receptor α (IL-4Rα) gene is recognized as a candidate gene for AR, encoding a receptor subunit shared by IL-4R and IL-13R, and involving several polymorphisms (24). Additionally, researchers have identified various single nucleotide polymorphisms (SNPs) associated with AR across different populations (25). Andiappan et al. discovered SNPs in the MRPL4 and BCAP genes, as well as associations within the HLA-DQ and NPSR1 loci, in a Singapore Chinese population. Similarly, studies in a Han Chinese population identified SNPs in the MRPL4 and TNF-α genes linked to AR (26). In European populations, studies reported associations of AR with variants in HLA, C11orf30, LRRC32, and rs2155219 (27). A study in a Korean population also found SNPs associated with AR: rs7275360, an intron variant on chromosome 21q21 linked to NCAM2, and rs698195 on 7q31.1, a region linked to chronic rhinosinusitis susceptibility (28). Additionally, research in ethnically diverse North American populations linked a locus on chromosome 7p21.1 near the FERD3L gene to AR (22). While these studies have identified various genetic variants associated with AR, they only partially explain the heritability of the condition. Emerging evidence suggests that environmental exposures may interact with epigenetic modifications, such as DNA methylation and histone modifications, influencing gene expression and contributing to both the development and severity of AR (29).

In recent years, the concept of trained immunity has provided new insights into the etiology of AR. Netea et al. first defined trained immunity as a form of long-term functional reprogramming of innate immune cells induced by exogenous or endogenous stimuli, leading to enhanced or diminished responses to subsequent non-specific stimuli (30). Trained immunity demonstrates that both microbial pathogens and non-microbial antigens, including dietary and other environmental factors, can influence innate immune regulation at both central and peripheral levels. The mechanisms primarily involve cellular epigenetic reprogramming and immune metabolic pathways. Different stimuli, such as β-glucans, LPS, or the Bacillus Calmette-Guérin vaccine, can induce distinct trained immunity programs, manifesting as either immune enhancement or tolerance. Specifically, after antigen stimulation, innate immune cells undergo reprogramming of pro-inflammatory and anti-inflammatory gene transcription through epigenetic modifications like histone H3K27ac, H3K4m3, and H3K4m1, as well as metabolic pathways involving glycolysis, glutamine, and cholesterol metabolism, altering their inflammatory and anti-inflammatory phenotypes upon subsequent stimulation (31–33).

Unlike the concept of a single microbial antigen in the hygiene hypothesis, the training immunity theory involves the effects of multiple endogenous and exogenous antigens on the immune system. This theory emphasizes the interplay of environmental, genetic, and metabolic factors, and how the type and timing of antigen stimulation affect immune tolerance or enhancement. Current research indicates that training immunity mechanisms play a significant role in the development and progression of infectious diseases, asthma, coronary atherosclerosis, neurodegenerative diseases, and tumor growth and metastasis. For example, a Western diet induces pro-inflammatory transcription and epigenetic reprogramming in mice prone to atherosclerosis, with these effects persisting even after diet modification to a standard diet (34). Familial hypercholesterolemia and severe coronary atherosclerosis patients exhibit significant levels of histone methylation modifications in monocytes and altered glycolytic metabolism, leading to an enhanced pro-inflammatory phenotype (35, 36). Recently, Machiels et al. demonstrated that trained alveolar macrophages, which retain the memory of prior viral infections, can offer protection against asthma induced by allergens (37). This study provides experimental evidence that training immunity not only supports but also extends the original hygiene hypothesis by showing how past infections can modulate immune responses to allergens.

While adaptive Th2-type immune responses form the basis of specific IgE responses in AR, innate immune cells involved in training immunity, such as monocytes, macrophages, and dendritic cells, also play a crucial role in the sensitization and reactivation processes of AR (38). Some studies have suggested that trained immunity may either promote or protect against AR. Jin et al. found through bidirectional two-sample Mendelian randomization analysis that *Coriobacteriia* and its subcategories (*Coriobacteriales* and *Coriobacteriaceae*) in the gut microbiota have a protective effect against AR, whereas *Victivallaceae* is a risk factor (39). Additionally, a 13-year follow-up study in a Finnish probiotic intervention cohort found that early-life gut microbiota is associated with the development of AR (40). These findings suggest that microorganisms may influence AR by modulating immune cells in the gut through trained immunity mechanism. Additionally, the severity of AR is influenced by the frequency and timing of allergen exposure, suggesting a role for training immunity. A cross-sectional survey in India showed that the severity and type of AR are related to allergen exposure, with healthcare workers exposed to dust mites and farmers exposed to pollen showing higher rates of moderate to severe AR (41). Tulic et al. found that the timing of endotoxin exposure after sensitization also affects IgE responses (42). Moreover, AR in children is more influenced by genetic factors and has greater plasticity in immune responses, making it more susceptible to regulation by training immunity. Therefore, introducing the concept of training immunity may help address the limitations of the hygiene hypothesis in explaining the onset and development of AR.

In addition, researchers have been exploring effective measures to prevent the onset of AR, with breastfeeding strategies being a key focus. Some earlier studies suggested a protective role of breastfeeding in preventing AR. For example, Codispoti et al. reported that prolonged breastfeeding among African American participants was associated with a reduced risk of AR at age three (43). Breastfeeding influences the gut microbiota, which in turn regulates immune homeostasis (44). In breastfed infants, the gut microbiota is dominated by *Bifidobacterium*, whereas in formula-fed infants, Bacteroides, Clostridium, and Enterobacteriaceae are more prevalent (45). Several clinical studies have reported that long-term supplementation with probiotics, such as *Lactobacillus casei*, *Lactobacillus rhamnosus*, and *Lactobacillus gasseri*, may alleviate AR symptoms in preschool children and help prevent IgE-mediated allergies and other allergic conditions (46–48). However, an increasing body of long-term cohort studies and cluster RCT evidence indicates that breastfeeding, along with probiotic supplementation, has no lasting effect on the prevention of AR (49–52). Notably, a Finnish cohort study involving 3,781 consecutively born children followed for five years, and the GINIplus study tracking 4,058 individuals until 20 years of age, found no significant association between breastfeeding and reduced AR risk (49, 53). Thus, while breastfeeding plays an important role in early immune modulation, there is currently insufficient evidence to support its preventive effect on AR. As a result, the 2022 German S3 Guideline for Allergy Prevention recommends breastfeeding, but this recommendation is not based on evidence related to AR prevention (54).

## 4 Diagnosis

**Figure 1:**
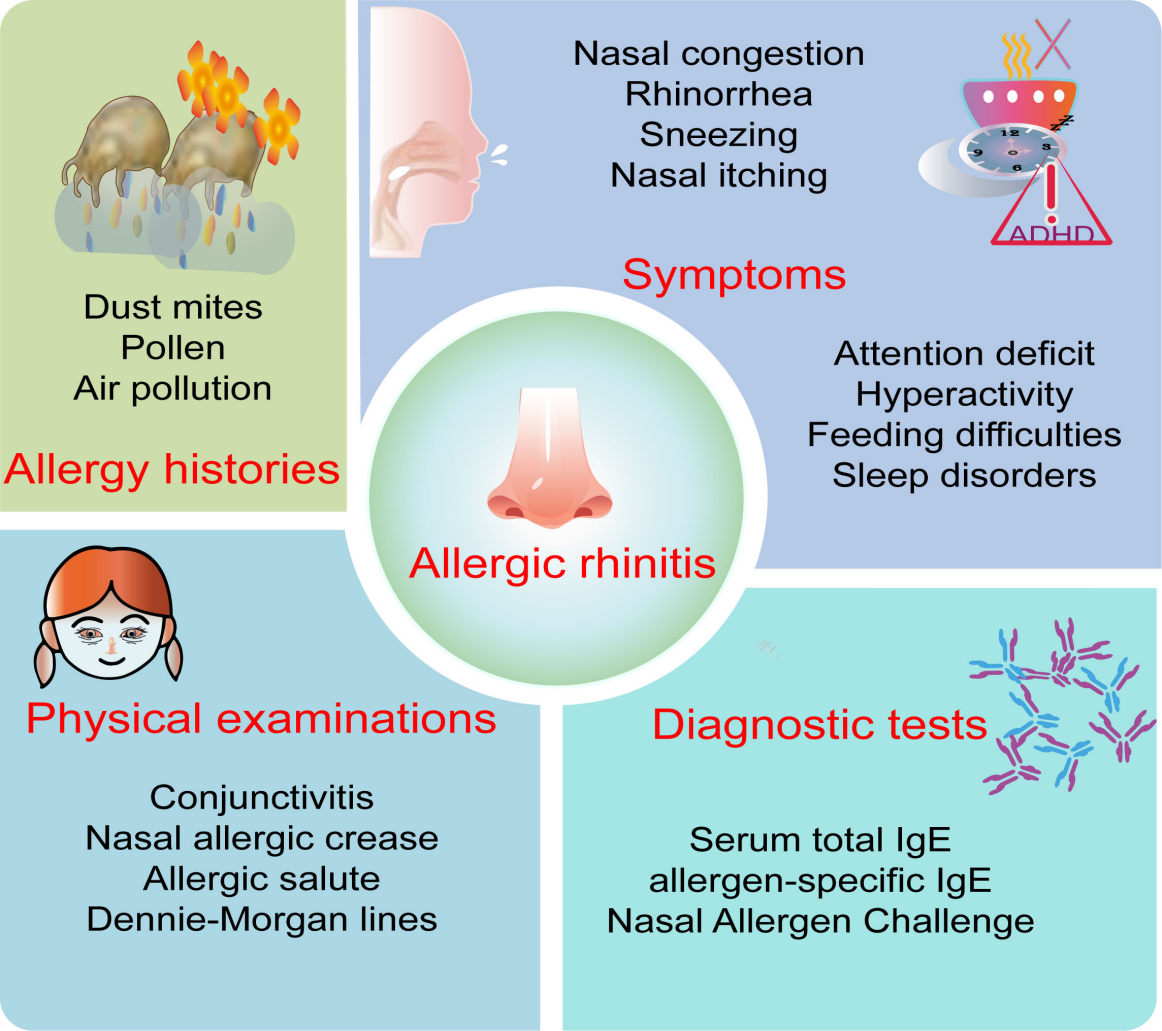
Key diagnostic points of allergic rhinitis in children. The key points for diagnosing allergic rhinitis in children include allergy history, symptoms, physical examination, and diagnostic test.

The diagnosis of AR is typically based on a detailed medical history, physical examination, and supported by specific allergen testing (**Figure 1**). To differentiate AR from other forms of rhinitis, physicians may employ additional tests, including nasal allergen challenge, CT scans, nasal nitric oxide measurements, nasal cytology, nasal culture, and nasal fluid β-transferrin analysis. However, due to the limited availability of allergen testing in infants and young children, along with inconsistent guidance from physicians across various disciplines, many diagnoses of AR in children rely solely on chief complaints, symptoms, or symptom scores.

### 4.1 Symptoms and signs

Caregivers’ descriptions of AR symptoms in children may not accurately reflect the severity of the condition compared to reports from adult patients. Typical symptoms of AR include sneezing, itching, rhinorrhea, and nasal congestion, with nasal congestion often being more pronounced at night and presenting bilaterally, unilaterally, or alternating between sides (55). Children may also exhibit mouth breathing and nighttime snoring, which, if chronic, can lead to facial developmental abnormalities and malocclusion. Ocular symptoms are more common in polysensitized patients and are correlated with the severity of nasal symptoms (3). Other symptoms may include itching of the palate, postnasal drip, cough, nosebleeds, hyperactivity, and attention deficit, while younger children may exhibit decreased appetite and feeding difficulties. Special signs such as conjunctivitis, nasal allergic crease, allergic salute, and Dennie-Morgan lines are also critical for the detection and diagnosis of pediatric AR. Interestingly, a study evaluating the value of history impression and physical examination in diagnosing AR found that the average sensitivity, specificity, positive predictive value, and negative predictive value of history impression were all higher than those of physical examination (56). Therefore, physicians must first obtain a detailed history of the patient’s symptoms and allergens and conduct a comprehensive physical examination.

### 4.2 Diagnostic tests

However, diagnosing AR in children is challenging, as its symptoms often overlap with those of upper respiratory tract infections, non-allergic rhinitis, and other conditions, which can mislead both families and healthcare providers. Therefore, appropriate diagnostic methods should be selected based on the specific needs for differential diagnosis or classification, including specific allergy tests, nonspecific allergy tests, and others.

Commonly used specific allergen detection methods include skin prick tests, serum specific IgE tests, and nasal provocation tests. While infants or young children may have difficulty cooperating with skin prick tests, blood tests can usually be performed. Among these methods, skin prick testing demonstrates high sensitivity and specificity (exceeding 80%) (57, 58). While skin prick testing can be used in children of all ages without contraindications (such as uncontrolled or severe asthma), it is important to note that infants may exhibit small wheals, false positives may occur in control groups, and the results are susceptible to influences such as the child’s immune status and procedural factors, requiring cautious interpretation (59). In cases where skin prick testing yields negative results but clinical suspicion for specific allergen sensitization remains high, intradermal testing may be considered. Compared to skin prick testing, serum allergen-specific IgE testing has advantages of being free from adverse reactions and less susceptible to interference from medication and skin conditions. Although some studies suggest that skin prick testing is generally more sensitive than serum allergen-specific IgE testing, comprehensive decision-making is still necessary, considering factors such as the child’s medication regimen, comorbidities, skin condition, and family preferences.

Although specific IgE measurement is crucial in allergy diagnosis, nasal allergen challenge (NAC) remains the preferred method when the clinical relevance of an allergen needs confirmation (60). NAC, also known as Nasal Allergen Provocation Test, is increasingly utilized in clinical practice for diagnosing local AR, identifying allergen components, and assessing AR treatment efficacy. Under standardized and controlled conditions, NAC can accurately reproduce nasal allergic responses. Studies have demonstrated that NAC is highly safe and reproducible in both adults and children, with minimal risk of systemic allergic reactions or bronchospasm (61). In a study by Eguiluz-Gracia et al. involving 518 children and 5830 adults undergoing NAC, only 4 adverse events were reported, with repeatability, positive predictive value, and negative predictive value of 97.32%, 100%, and 92.91%, respectively (61, 62).

NAC results can be evaluated by assessing clinical symptoms through total nasal symptom score (TNSS) and Visual Analogue Scale (VAS), or by measuring nasal patency using peak nasal inspiratory flow (PNIF), rhinomanometry, and acoustic rhinometry (63). Currently, worsening subjective symptoms and increased objective nasal resistance are both considered as criteria for NAC positivity, either separately or in combination. For instance, Sasiwimon et al. reported that NAC is considered positive if any one of the following three criteria is met: nasal airway resistance increases by at least 20% from baseline, TNSS changes by at least 3 points; PNIF decreases by at least 20% from baseline, TNSS changes by at least 3 points; or nasal airway resistance increases by at least 40% from baseline, regardless of TNSS change (64).

Since children may be less accurate in describing subjective symptoms or may not cooperate well with nasal resistance tests, the European Academy of Allergy and Clinical Immunology (EAACI) recommends NAC for children over five years old (65). However, age is not an absolute contraindication; the child’s ability to understand and cooperate with the procedure should be considered. Additionally, potential false negatives due to medications (e.g., antihistamines, corticosteroids) or false positives due to environmental allergens should be ruled out before conducting and interpreting NAC results (66).

Additionally, although total serum IgE levels and eosinophilia are often used in adults as screening tests for allergies, their relatively low sensitivity precludes their routine use in diagnosing AR. Given that serum total IgE levels are frequently normal in AR children, and interference from other allergic factors is likely, the diagnostic value of serum total IgE in AR is generally considered limited (67). In recent years, biomarkers such as nasal nitric oxide (nNO) measurement and eosinophil detection in nasal smears have also been commonly used in AR. A large-scale study involving 173 patients who underwent nasal sinus CT scans and 46 normal controls revealed that patients with AR exhibited significantly higher levels of nNO compared to those with non-allergic rhinitis. The determination of nNO levels proved to be an effective means of distinguishing between these two phenotypes of rhinitis (68). Dynamic monitoring of nNO may contribute to disease assessment and monitoring (69). Measuring nNO has advantages such as being non-invasive and convenient. However, due to the lack of a normal reference value standard for exhaled NO in the nasal cavity, its clinical diagnostic value cannot be determined at present. Other emerging biomarkers for AR, such as nasal mucosal osteoprotegerin, can provide additional information on inflammation and remodeling. The expression of CD203c on the surface of eosinophils may be associated with the temporal characteristics of AR symptoms (70). Furthermore, nasal CT scans have certain value in exclusive diagnosis. However, due to the presence of ionizing radiation, the indications for its use in children should be carefully considered.

### 4.3 Ongoing clinical trials in AR diagnostics

Currently, few ongoing clinical trials on AR diagnostics include pediatric populations, as cataloged in ClinicalTrials.gov and the WHO International Clinical Trials Registry Platform (**Table 1**). One trial investigates the application of multimodal data, integrating voice and facial recognition, to enhance early screening efficiency via artificial intelligence. Another focuses on molecular allergen component-resolved diagnostics to refine personalized immunotherapy. These studies hold promise for advancing diagnostic accuracy and optimizing treatment outcomes in AR.

**Table 1:** Ongoing clinical trials in AR Diagnostics.

Trial No.	Study Title	Study Type	Study Status	Inclusion agemin	Inclusion agemax	Country
NCT06474923	Multimodal Data-assisted Primary Screening for Allergic Rhinitis Based on Voice Recognition and Face Recognition	Observational	Recruiting	8 Years	80 Years	China
NCT05448066	Molecular Allergen Component Resolved Diagnosis to Decide Immunotherapy	Interventional	Recruiting	5 Years	No Restriction	Portugal

### 4.4 Comorbidities of AR

Beyond its primary symptoms and clinical manifestations, AR is frequently associated with other allergic conditions, including asthma, atopic, conjunctivitis, and dermatitis. Over 80% of asthma patients are also affected by AR, while approximately 10-40% of AR patients have asthma (71). The inflammatory processes in the nasal mucosa of AR and the bronchial mucosa of asthma share similar characteristics, such as pro-inflammatory mediators, T-helper cell type 2 (Th2) cytokines, chemokines, and adhesion molecules, supporting the concept of “one airway, one disease” (72). Evidence from cohort studies indicates that early childhood AR is a significant predictor of the persistence of asthma in children (73). The comorbidity of AR with asthma or atopic dermatitis may be attributed to shared genetic polymorphisms and allergen-triggered pathogenic mechanisms. Recent research involving integrated transcriptomic analysis of over 1,200 participants has identified a specific gene signature linked to the multimorbidity of AR, asthma, and atopic dermatitis. The study consistently found eight overexpressed genes (e.g., CLC, IL5RA, SIGLEC8) across these conditions, indicating a shared biological foundation for their frequent co-occurrence (74). Additionally, a meta-analysis has revealed a significant association between AR and symptoms of attention-deficit hyperactivity disorder in children, including total symptom scores, hyperactivity/impulsivity, and inattention (75). AR is also the most common non-rheumatic comorbidity in Juvenile Idiopathic Arthritis (76). The multimorbidity associated with AR exacerbates the overall disease burden in children and has long-term adverse effects on their health. Therefore, it is essential to assess comorbidities in children with AR and consider combined treatment strategies when necessary.

## 5 Classification

Effective management of AR requires dynamic decision-making, particularly in children, where classification is often based on the onset, timing, and severity of symptoms (77). Traditionally, AR was classified into perennial, seasonal, and occupational forms, depending on allergen exposure (78). Seasonal AR is typically triggered by outdoor allergens like pollen, while perennial AR is associated with indoor allergens such as dust mites and pet dander (79, 80). However, due to the year-round presence of certain plant pollens, variations in pollination seasons across different regions, the existence of patients sensitive to perennial allergens but exhibiting only short-term symptoms, the high prevalence of polysensitized patients, and asymptomatic allergic individuals, the ARIA (Allergic Rhinitis and its Impact on Asthma) guidelines proposed a new classification system for AR (71). This classification subdivides AR based on symptom frequency into ‘intermittent’ and ‘persistent’ forms, with persistence defined as symptoms occurring more than four days per week for at least four weeks. Additionally, ARIA classifies AR into mild, moderate, or severe categories based on the severity of symptoms and their impact on quality of life.

The ARIA classification system is currently recognized and widely adopted by most countries (81, 82). However, there is still no unified standard for assessing the severity of AR. Clinically, severity is often evaluated using TNSS, VAS, nasal obstruction measurements, and olfactory assessments (71). Evidence suggests that using VAS to assess AR severity is not influenced by treatment or allergy diagnostic tests (83). The ARIA classification based on frequency and severity aids in AR management, with moderate to severe persistent AR showing a stronger association with respiratory comorbidities and sensitization compared to mild AR (84). Therefore, treatment strategies based on the ARIA classification have practical significance. For instance, the 2022 Chinese expert consensus on the stepwise treatment of pediatric AR employs a straightforward and quantifiable VAS to score AR, distinguishing between mild (VAS < 5) and moderate-severe (VAS ≥ 5) cases (85). This scoring system further categorizes AR into sneezing/rhinorrhea-predominant and nasal obstruction-predominant types, each with corresponding stepwise treatment plans and assessment methods.

Recent studies have identified a subset of AR patients who test positive in NAC but show no sensitization in skin prick tests or serum-specific IgE assays, a condition known as local allergic rhinitis (LAR) (86). Evidence suggests that while LAR in children is less likely to progress to systemic allergic diseases, it may worsen over time and serves as a risk factor for asthma (87). The diagnosis of LAR relies on a positive NAC response to one or more allergens; however, both adult and pediatric LAR remain underdiagnosed.

## 6 Treatment and health management

The management of AR in children emphasizes a comprehensive step-by-step strategy, which includes environmental control, medication therapy, immunotherapy, and health management (88). Strategies may involve allergen avoidance, patient education, antihistamine treatment, saline nasal irrigation, and specific immunotherapy. In moderate to severe cases, combination therapy using corticosteroids and leukotriene receptor antagonists (LTRA) might be necessary. In situations where mild AR is not well-controlled, an escalation of the treatment regimen is warranted, while for adequately managed moderate to severe cases, a gradual reduction in treatment intensity can be considered.

### 6.1 Environmental control

Clinical epidemiological studies have shown that environmental air pollution, dust mites, and pets can promote the development of AR. A study in Changchun, China, demonstrated that for each standard deviation increase in PM2.5 pollutants, the number of visits by AR patients increased by 10.2% (89). Some studies support that environmental control can effectively reduce allergen exposure and improve the health of children with AR. For example, for AR caused by dust mites, acaricides and high-efficiency particulate air filters have specific therapeutic effects, while the removal of pets also shows some effectiveness for certain patients with AR (1). However, some studies indicate that although environmental control measures reduce allergen exposure levels, their effect on alleviating symptoms or improving the quality of life of AR patients is limited (90).

Another environmentally focused strategy that has gained attention in recent years is allergen barrier agents, primarily including nasal sprays and nasal ointments. These formulations create a mechanical barrier to avoid allergen contact with the nasal mucosa, thereby alleviating allergy symptoms. Multiple studies have shown that allergen barrier agents, whether used as monotherapy or in combination with other medications for pediatric AR, significantly reduce symptom scores, improve quality of life, and do not increase the incidence of adverse reactions (91–93). Although the use of allergen barrier agents in infants and toddlers may be influenced by nasal medication compliance, if this form of physical barrier treatment can mitigate the side effects of drug therapy, it is likely to be more accepted by children and parents. However, existing studies have small sample sizes and short follow-up periods, so the long-term efficacy of allergen barrier agents requires further validation through large-sample, long-term clinical studies.

### 6.2 Medication treatment

Considering factors such as efficacy and treatment duration, pharmacotherapy remains the most widely accepted treatment approach currently. Pharmacological interventions are primarily selected based on the frequency and severity of symptoms in AR, encompassing oral or intranasal H1 antihistamines, intranasal corticosteroids (INCS), and fixed combinations of intranasal H1 antihistamines and corticosteroids (94).

H1-antihistamines are commonly used for patients with mild symptoms or those who are averse to INCS therapy. This includes second-generation oral H1-antihistamines with lower sedation levels (such as desloratadine, loratadine, cetirizine, levocetirizine, and rupatadine) and non-sedating H1-antihistamines (such as fexofenadine and bilastine). Oral H1-antihistamines, administered once daily, are rapidly effective and can be used as a single agent intermittently or continuously, effectively controlling symptoms in many pediatric patients while also offering the advantage of lower cost. However, the potential systemic side effects, including sedation, dry eye syndrome, and urinary retention, should not be overlooked. Previous research has confirmed the safety of desloratadine, levocetirizine, and levocetirizine in children aged 6 months and older, as well as the safety of loratadine in children aged 2 years and older. The latest evidence also affirms the efficacy and safety of rupatadine (with dual affinity for H1 receptors and platelet-activating factor receptors) in children aged 2 years and older (95). For children aged 6 years and older with seasonal or perennial AR, intranasal antihistamines are also a viable option. Currently, intranasal antihistamines approved by the U.S. FDA, including azelastine and olopatadine, are more effective for nasal congestion and have a faster onset of action than oral antihistamines, but they may increase the risk of local side effects, such as epistaxis (96).

INCS remains the most effective monotherapy for treating AR and are commonly employed as the first-line treatment option for patients with persistent or moderate-to-severe symptoms. Frequently used medications include beclomethasone, budesonide, ciclesonide, fluticasone propionate, fluticasone furoate, mometasone furoate, and triamcinolone acetonide. INCS not only effectively controls nasal symptoms in AR patients but also demonstrates efficacy in managing allergic ocular symptoms (57). The therapeutic effectiveness of regularly used INCS surpasses that of oral antihistamines, especially in alleviating nasal congestion. Additionally, the addition of oral antihistamines to INCS treatment does not typically enhance therapeutic outcomes. Recent evidence from randomized controlled trials suggests that, for patients with moderate-to-severe AR, both as-needed and regular use of INCS yield similar improvements in nasal symptom scores and Rhinitis Life Quality-36 questionnaire scores, with the exposure dose for as-needed use being only half of that for regular use (97). Systematic reviews and meta-analyses further support the comparable efficacy of as-needed INCS use to regular use (98). Given the absence of systemic absorption and concerns about systemic adverse reactions, the most common local adverse reactions associated with INCS include nasal irritation, stinging, and nosebleeds, which can be prevented by directing the spray away from the nasal septum.

When the efficacy of monotherapy is suboptimal, consideration may be given to the fixed combination of intranasal antihistamines and INCS), such as fluticasone propionate-azelastine and mometasone-olopatadine. Studies have demonstrated that the fixed combination of INCS and intranasal H1-antihistamines is more effective than individual drug administration and is well-tolerated (99). In the context of combination therapy, caution should be exercised to avoid the use of combinations without additional benefits, with careful consideration of potential side effects and interactions associated with drug co-administration.

In addition, montelukast, a LTRA, is commonly used to treat pediatric AR. The U.S. Food and Drug Administration (FDA) has approved its use for seasonal AR in children aged two and above and perennial AR in children aged six months and above. Children using montelukast generally exhibit good tolerability, but occasional neurobehavioral events may occur. A study from Korea showed an increased risk of neurobehavioral events in adolescents (12-18 years) and young adults (19-30 years) using LTRA in patients with asthma or AR, while no such increase was observed in children (3-11 years) (100). Although some children with concomitant asthma may benefit, overall, its efficacy is not superior to oral H1-antihistamines or INCS. Therefore, there is currently no evidence supporting the routine use of LTRA for the treatment of pediatric AR.

### 6.3 AIT

Due to concerns about long-term medication and drug side effects, there is a growing preference for AIT that gradually introduces allergens to enhance tolerance in pediatric patients, thereby reducing or eliminating allergic reactions. The goal of AIT is to alleviate allergy symptoms, improve quality of life, modify the natural course of the disease, and provide lasting relief from allergies over the long term. Evidence suggests that AIT can also prevent new sensitizations and reduce the risk of asthma development in AR patients (101). AIT treatment can be considered in cases with allergen-specific IgE positivity.

The typical duration of AIT is 3-5 years, including an induction phase and a maintenance phase. During the induction phase, allergen doses are gradually increased to establish tolerance. After reaching the maintenance phase, regular administration of maintenance doses of allergens is required to sustain tolerance. Currently, AIT mainly involves subcutaneous immunotherapy and sublingual immunotherapy. Real-world evidence confirms the effectiveness of subcutaneous or sublingual immunotherapy in pediatric AR, with outcomes possibly superior in children compared to adults, as demonstrated by a significant reduction in prescription medication use for AR in children evaluated over 3-9 years post-treatment (102). Sublingual immunotherapy, in comparison to subcutaneous immunotherapy, exhibits higher compliance and fewer, milder adverse reactions (103). Studies have shown that increasing the immunization dose within a certain range can enhance the effectiveness of sublingual immunotherapy (104).

In recent years, researchers have explored some relatively short-duration alternatives, such as intralymphatic immunotherapy, epicutaneous immunotherapy, and intradermal immunotherapy, showing some effectiveness and relative safety. However, there is still insufficient evidence to support their superiority over subcutaneous or sublingual immunotherapy in children (103). Nevertheless, AIT may lead to serious adverse reactions, such as systemic allergic reactions, and should be conducted under the guidance of a physician. Additionally, for children in the developmental stage, careful consideration is needed due to the potential adverse impact of AIT on delaying symptom control.

### 6.4 Other treatment options

Researchers have been continuously exploring more convenient and less side-effect treatment methods for AR. Saline irrigation can remove some allergens and inflammatory mediators, offering advantages such as safety and convenience, and it is easily accepted by families of older children and infants. A Cochrane systematic review showed that isotonic or hypertonic saline irrigation effectively reduces the severity of AR symptoms and enhances the effectiveness of pharmacotherapy (105).

Moreover, increasing evidence in recent years supports the regulatory role of traditional Chinese medicine in treating AR. For instance, several studies revealed the efficacy and safety of Xiao Qing Long Tang in treating AR (92, 106). However, more robust evidence is needed for the use of traditional Chinese medicine in children with AR. Additionally, careful consideration is required regarding children’s tolerance of the unpleasant taste of herbal medicine and the potential for drug-induced liver and kidney damage. Some studies suggest that acupuncture and moxibustion can improve AR symptoms (107, 108). However, their application in children is rare.

### 6.5 Health management

Ensuring the optimal effectiveness of various AR treatment measures relies on long-term adherence and the standardization of treatment. Research indicates that the compliance of patients undergoing AIT during a 12-month follow-up period is significantly higher than those with a 3-month or 6-month follow-up period (65). Therefore, effective health management is crucial for improving treatment compliance and ensuring the efficacy of AR therapy in children.

**Figure 2:**
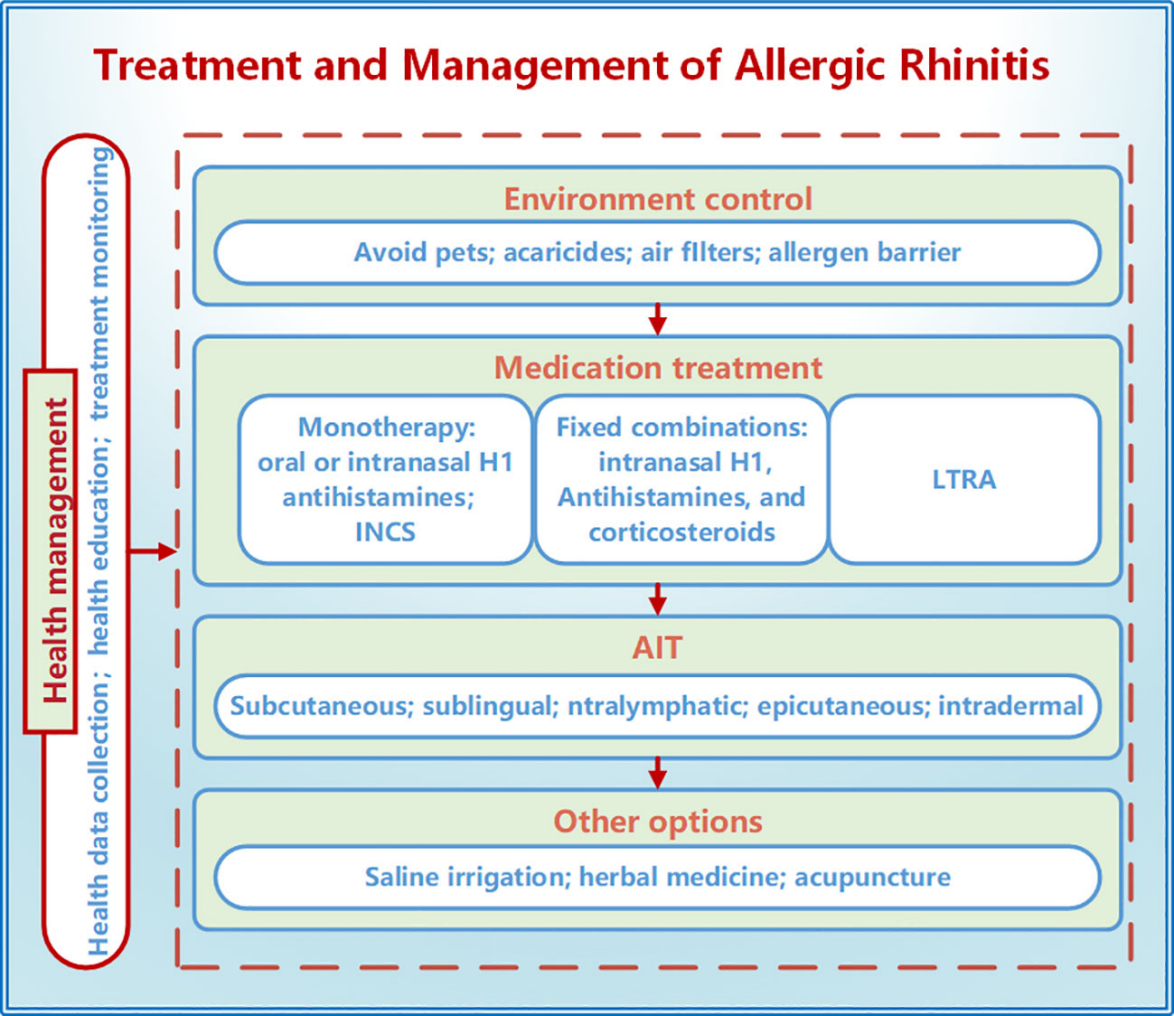
Treatment and management of allergic rhinitis in children. The treatment methods for allergic rhinitis in children include environmental control, medication treatment, AIT, saline irrigation, traditional Chinese medicine, and acupuncture. Health management can improve treatment adherence and efficacy through measures such as data collection, health education, and treatment management. INCS, intranasal corticosteroids; LTRA, leukotriene receptor antagonists; AIT, allergen-specific immunotherapy.

With the widespread adoption of smartphones globally and breakthroughs in artificial intelligence technology, there is a promising prospect for the development and promotion of mobile health (mHealth) applications that enable real-time collection, analysis, and feedback of patient data (109). More health management measures are anticipated to enhance treatment adherence, improve quality of life, and disease prognosis for children with AR through behavior change strategies such as reminders, consultations, reinforcement, or education (**Figure 2**).

ARIA proposed the use of mobile communication technology to develop and validate information technology tools that strengthen self-medication management for AR patients and facilitate shared decision-making with healthcare professionals (110). Currently, the Mobile Airways Sentinel Network (MASK) stands as the most influential AR mHealth tool. MASK is a patient-centered information and communication technology system that includes a treatment list, which contains country-specific medications, and a visual analogue scale to assess AR control, sleep, work efficiency, etc. The study, conducted among over 9,000 users from 22 countries/regions as part of the MASK initiative, reveals poor treatment adherence among patients with AR. This includes self-medication, on-demand treatment when symptoms are not optimally controlled, switching medications to gain control when symptoms are unmanageable, and non-compliance with medical guidelines or physician prescriptions (111–113).

The Control of Allergic Rhinitis/Asthma Test (CARAT) is a powerful tool for screening AR diagnosis and assessing disease control (114). CARAT consists of a questionnaire regarding symptoms, sleep, activity, medication usage, and other aspects over the past four weeks. It summarizes the patient’s clinical condition and supports shared decision-making among patients, doctors, and the healthcare team. These tools have been used in clinical research and practice, enabling personalized and real-time assessment for patients. A study involving 643 patients revealed a significantly worse RHINASTHMA questionnaire total and subdomain scores and symptom control in patients with AR + asthma than in patients with AR alone (115). Therefore, the importance of implementing health management and comprehensive management through mHealth to achieve optimal care and ensure the effectiveness of AR treatment cannot be overstated.

### 6.6 Ongoing clinical trials in AR treatment and management

Data from ClinicalTrials.gov and the WHO International Clinical Trials Registry Platform (**Table 2**) reveal that current clinical trials on pediatric AR predominantly target immunotherapy, combination therapies, and alternative treatments. For instance, a fixed-dose nasal spray combining mometasone and azelastine is being tested for its enhanced anti-inflammatory and decongestant effects. Natural therapies, such as black seed spray and laser acupuncture, are also under investigation, offering patients additional treatment options and aiming to improve quality of life.

**Table 2:** Ongoing clinical trial overview in AR treatment and management.

Trial No.	Study Title	Study Type	Study Status	Inclusion agemin	Inclusion agemax	Phase (N/A = Not Applicable)	Country	Treatment/ Management
NCT06525597	Study of Stapokibart Injection in Patients With Allergic Rhinitis	Interventional	Not recruiting	12 Years	65 Years	Phase 2	China	Treatment
NCT06523478	Intratonsillar Immunotherapy for Allergic Rhinitis	Interventional	Recruiting	5 Years	65 Years	N/A	China	Treatment
NCT06272409	Efficacy and Safety of DEP114 in the Treatment of Moderate to Severe Persistent Allergic Rhinitis in Children.	Interventional	Not yet recruiting	6 Years	11 Years	Phase 3	Unpublished	Treatment
NCT06104332	PMCF to Assess Real-life Usage Effectiveness, Safety and Patient Satisfaction of a Range of Hypertonic Seawater-based Decongestant Nasal Sprays	Observational	Recruiting	3 Years	No Restriction	N/A	France	Treatment
NCT06021912	e-ITAG Allergen Immunotherapy in the Management of Allergic Asthma	Observational	Recruiting	5 Years	65 Years	N/A	Tunisia	Treatment
NCT05887843	Study to Compare the Pharmacokinetics of Fixed-Dose Combination of Mometasone + Azelastine Nasal Spray to Mometasone and Azelastine Nasal Sprays in Adolescents and Young Adults With Seasonal Allergic Rhinitis	Interventional	Not recruiting	12 Years	24 Years	Phase 1	Canada	Treatment
NCT05720455	Study to Assess Safety and Efficacy of Fexofenadine Hydrochloride (HCL) + Pseudoephedrine HCL Fixed Dose Combination in Indian Male and Female Participants With Allergic Rhinitis (AR) Who Are 12 Years and Above	Interventional	Not recruiting	12 Years	No Restriction	Phase 4	Unpublished	Treatment
NCT05684380	Efficacy and Safety of MAZ-101 in the Treatment of Persistent Allergic Rhinitis (PER)	Interventional	Not recruiting	12 Years	No Restriction	Phase 3	Unpublished	Treatment
NCT05668390	Safety and Efficacy of STALORAL庐 Birch 300 IR in a Paediatric Population With Birch Pollen-induced ARC w/o Asthma	Interventional	Active, Not recruiting	5 Years	17 Years	Phase 3	Sweden	Treatment
NCT05641272	Clinical Trial to Evaluate the Efficacy and Safety of Polymerized, Mannan-Conjugated Dermatophagoides Allergen Extract	Interventional	Not recruiting	12 Years	60 Years	Phase2/ Phase 3	Argentina	Treatment
NCT05637710	Parallel, Double-dummy, Superiority Study Levocetirizine/Pseudoephedrine x Zina for Allergic Rhinitis in Brazil	Interventional	Not recruiting	12 Years	65 Years	Phase 3	Brazil	Treatment
NCT05553483	Effect of Laser Acupuncture Alone or Combined With Pranayama Exercise on Inflammation in Allergic Rhinitis	Interventional	Recruiting	12 Years	17 Years	N/A	Egypt	Treatment
NCT05494346	Safety and Performance Assessment of the Decongestant Seawater Spray Pocket Valve Enriched With Essential Oils in Patients With Acute Rhinitis Associated With Nasal Obstruction	Interventional	Recruiting	12 Years	No Restriction	N/A	France	Treatment
NCT05476484	Comparative Real World Effectiveness of SQ Sublingual Immunotherapy (SLIT)-Tablets vs. Controls in Allergic Rhinitis and Asthma	Observational	Active, Not recruiting	5 Years	No Restriction	N/A	Denmark	Treatment
NCT05450289	The Efficacy of Nigella Sativa in Children With House Dust Mite-Induced Respiratory Allergy Receiving Immunotherapy	Interventional	Unknown status	2 Years	17 Years	N/A	Indonesia	Treatment
NCT05395689	Efficacy and Safety Assessment of Beltavac庐 With Polymerized Extract of HDM	Interventional	Recruiting	12 Years	65 Years	Phase 3	Spain	Treatment
NCT05299086	As Needed Versus Regular Intranasal Corticosteroid in Children With Perennial Allergic Rhinitis	Interventional	Recruiting	6 Years	18 Years	N/A	Thailand	Treatment
NCT05234580	Safety and Efficacy Study of PA9159 Nasal Spray for the Treatment of Seasonal Allergic Rhinitis	Interventional	Not recruiting	12 Years	No Restriction	Phase 2	China	Treatment
NCT05214911	Fixed Dose Combination of Desloratadine / Prednisolone in the Treatment of Moderate Severe Allergic Rhinitis in Children	Interventional	Recruiting	6 Years	12 Years	Phase 3	Brazil	Treatment
NCT05186025	Tyrosine Allergoid Paediatric and Adult Study	Observational	Active, Not recruiting	5 Years	No Restriction	N/A	Germany	Treatment
NCT04891237	Efficacy and Safety Evaluation for the Treatment of Allergy Against Grass and Olive Pollen	Interventional	Recruiting	12 Years	65 Years	Phase 3	Spain	Treatment
NCT03872219	Biodiversity Intervention and Atopic Sensitization	Interventional	Not yet recruiting	0 Years	1 Month	N/A	Finland	Treatment
TCTR20240318004	Efficacy of Xyloglucan nasal spray in allergic rhinitis children	Interventional	Recruiting	6 Years	18 Years	Phase 3	Thailand	Treatment
KCT0006625	Combined Korean medicine therapies in children with allergic rhinitis: A multi-center, observational explanatory registry trial.	Observational	Recruiting	0 Years	12 Years	NA	Korea	Treatment
IRCT20220411054489N1	The efficacy of Black seed spray in the treatment of Nasal allergic inflammation	Interventional	Recruiting	3 years	15 years	Phase 2/ Phase 3	Iran	Treatment
EUCTR2021-004050-31-PL	Efficacy and safety of the combination Mometasone furoate + Azelastine hydrochloride nasal spray in the treatment of seasonal allergic rhinitis	Interventional clinical trial of medicinal product	Not recruiting	12 years	65 years	Phase 3	Moldova;Poland;Bulgaria;Germany	Treatment
EUCTR2020-004372-17-DE	Clinical study to investigate the effect and safety of a tree pollen immunotherapy tablet in children and teenagers with a birch pollen allergy	Interventional clinical trial of medicinal product	Not recruiting	4 years	18 years	Phase 3	Canada;Austria;Netherlands;Russia;Belgium;Hungary;Poland;Denmark;Slovakia;Lithuania;Germany	Treatment
EUCTR2020-000446-34-SK	A clinical trial studying the safety of the house dust mite tablets in adolescents with allergic rhinitis/rhinoconjunctivitis	Interventional clinical trial of medicinal product	Not recruiting	1 years	17 years	Phase 3	Czech;Slovakia;Germany	Treatment
CTRI/2024/07/070682	A Clinical Study To Ascertain Role of Homoeopathic Medicines in Allergic Rhinitis in Children Using by Use of Synthesis Repertory.	Interventional	Not recruiting	2 years	18 years	Phase 2	India	Treatment
CTRI/2024/07/070045	Homoeopathic Management of Allergic Rhinitis in Children	Interventional	Not recruiting	0 Years	17 Years	Phase 2	India	Treatment
CTRI/2022/03/041250	Regulating the immunity (Immunomodulatory) in children with wheeze by using homeopathic drug arsenicum album	Interventional	Not recruiting	0 Years	17 Years	Phase 4	India	Treatment
CTRI/2020/02/023517	Effect of Shatyadhi churna in the managment of allergic rhinitis (vataja pratisyaya ) among children.	Interventional	Not recruiting	6 Years	16 Years	Phase 2/ Phase 3	India	Treatment
CTIS2023-508520-36-00	Long-term clinical trial of PQ Grass in paediatric subjects with seasonal allergic rhinitis and/or rhinoconjunctivitis induced by grass pollen	Interventional clinical trial of medicinal product	Not recruiting	0 Years	17 Years	Phase 3	United States; Bulgaria;Czechia;Germany;Lithuania;Romania;Poland;Slovakia	Treatment
CTIS2023-508013-16-00	A prospective, randomized, double-blind placebo-controlled multicentre study with mannan-conjugated birch pollen allergoids administered subcutaneously to adolescent and adult patients with birch pollen-induced allergic rhinitis or rhinoconjunctivitis.	Interventional clinical trial of medicinal product	Not recruiting	0 Years	64 Years	Phase 3	Germany;Poland	Treatment
ChiCTR2400084131	A prospective, randomized, double-blind, parallel controlled clinical trial of spleen aminopeptide oral solution for the prevention and treatment of seasonal allergic rhinitis in children	Interventional	Not recruiting	4 Years	12 Years	Phase 4	China	Treatment
ChiCTR2000040868	Clinical efficacy and mechanism study of pressing needle in the treatment of children with allergic rhinitis	Interventional	Recruiting	6 Years	14 Years	N/A	China	Treatment
ChiCTR2000033160	A real world study of Guizhi and Longgu Muli Decoction in children with seasonal allergic rhinitis (Lung and Spleen Deficiency Syndrome)	Observational	Not recruiting	3 Years	14 Years	Phase 0	China	Treatment
ChiCTR2000031175	Application of probiotics in adjuvant treatment of infant allergic rhinitis: a randomized controlled study	Interventional	Not recruiting	0 Years	6 Years	Phase 4	China	Treatment
NCT06151938	Evaluate Measurement Instruments Relevance in Assessing Effectiveness of ACARIZAX® in House Dust Mite Allergic Rhinitis	Observational	Recruiting	12 Years	65 Years	N/A	China	Management
NCT05655858	Observational Study of Allergic Rhinitis in Children	Observational	Recruiting	2 Years	18 Years	N/A	France	Management
NCT04927689	Evaluation of an Environmental Counsellor's Home Stay in Children Treated for Asthma or Allergic Rhinitis Via a Standardized Medical Questionnaire Randomized Controlled Superiority Trial in Parallel Arms, Multicentric (EvalPCE)	Observational	Recruiting	6 Years	18 Years	N/A	France	Management
NCT03075917	Validation of an Allergic Rhinitis Control Test in Children	Interventional	Recruiting	5 Years	11 Years	N/A	France	Management

In the management of AR, there is a growing emphasis on refining personalized treatment strategies for children. One trial is evaluating the efficacy of ACARIZAX^®^ in children with house dust mite-induced AR, aiming to optimize efficacy assessment metrics to enhance therapeutic outcomes. A French study is analyzing real-world management of pediatric AR, while the Evaluation of an Environmental Counsellor’s Home Stay trial explores the role of environmental counseling in AR caring. Furthermore, a novel allergic rhinitis control test is undergoing validation, aiming to enhance disease monitoring precision.

Notably, an increasing number of clinical trials are incorporating real-world data to evaluate the effectiveness and patient satisfaction of these new therapies in practical settings. In pediatric AR, the trend toward personalized and precision-based treatment strategies is gaining momentum, with the potential to significantly improve both short- and long-term patient outcomes.

## 7 Conclusion

The rising prevalence of pediatric AR highlights the complex interaction between genetics and environmental factors. The concept of training immunity helps explain how environmental exposures influence immune responses and AR development. Combining environmental data with multi-omics can lead to better prevention and early identification of high-risk pediatric AR cases.

Despite advances in allergen testing and molecular diagnostics, choosing the right diagnostic methods remains challenging for clinicians. Optimizing diagnostic protocols for different ages and symptoms, and improving family education to prevent self-diagnosis, are essential.

For treatment, a stepwise approach tailored to age, AR type, and severity is recommended. Environmental control, saline irrigation, pharmacotherapy, immunotherapy, and traditional Chinese medicine can enhance quality of life. Developing long-term management strategies and personalized treatments is crucial. mHealth offers a promising tool for refining and optimizing AR management based on real-time feedback.
